# The seven-transmembrane receptor Gpr1 governs processes relevant for the antagonistic interaction of *Trichoderma atroviride* with its host

**DOI:** 10.1099/mic.0.052035-0

**Published:** 2012-01

**Authors:** Markus R. Omann, Sylvia Lehner, Carolina Escobar Rodríguez, Kurt Brunner, Susanne Zeilinger

**Affiliations:** 1 Research Area Molecular Biotechnology and Microbiology, Center for Chemical Engineering, Vienna University of Technology, Gumpendorferstrasse 1a, Wien, Austria; 2 Department for Agrobiotechnology IFA-Tulln, Institute of Analytical Chemistry, Konrad-Lorenz-Straße 20, Tulln, Austria

## Abstract

Mycoparasitic *Trichoderma* species are applied as biocontrol agents in agriculture to guard plants against fungal diseases. During mycoparasitism, *Trichoderma* directly interacts with phytopathogenic fungi, preceded by a specific recognition of the host and resulting in its disarming and killing. In various fungal pathogens, including mycoparasites, signalling via heterotrimeric G proteins plays a major role in regulating pathogenicity-related functions. However, the corresponding receptors involved in the recognition of host-derived signals are largely unknown. Functional characterization of *Trichoderma atroviride* Gpr1 revealed a prominent role of this seven-transmembrane protein of the cAMP-receptor-like family of fungal G-protein-coupled receptors in the antagonistic interaction with the host fungus and governing of mycoparasitism-related processes. Silencing of *gpr1* led to an avirulent phenotype accompanied by an inability to attach to host hyphae. Furthermore, *gpr1-*silenced transformants were unable to respond to the presence of living host fungi with the expression of chitinase- and protease-encoding genes. Addition of exogenous cAMP was able to restore host attachment in *gpr1*-silenced transformants but could not restore mycoparasitic overgrowth. A search for downstream targets of the signalling pathway(s) involving Gpr1 resulted in the isolation of genes encoding e.g. a member of the cyclin-like superfamily and a small secreted cysteine-rich protein. Although silencing of *gpr1* caused defects similar to those of mutants lacking the Tga3 Gα protein, no direct interaction between Gpr1 and Tga3 was observed in a split-ubiquitin two-hybrid assay.

## Introduction

The genus *Trichoderma* includes species that are potent mycoparasites, able to attack and lyse plant pathogens such as *Rhizoctonia solani*, *Botrytis cinerea*, *Sclerotium sclerotiorum*, *Pythium* spp. and *Fusarium* spp. *Trichoderma* mycoparasitism involves a combination of host recognition, attachment to and coiling around the host hyphae, and the secretion of antibiotic metabolites and cell-wall-degrading enzymes (e.g. Elad *et al.*, 1983; Baker, 1987; Chet *et al.*, 1998; Hjeljord & Tronsmo, 1998). In addition, *Trichoderma* strains used as biocontrol agents are able to induce defence responses in plants (Harman *et al.*, 2004).

Production of hydrolytic enzymes, such as chitinases, glucanases and proteases, plays a major role in mycoparasitism by *Trichoderma* as these enzymes degrade the cell wall of the host fungus to enable penetration of the mycoparasite (Hjeljord & Tronsmo, 1998). Besides the living host, isolated components of the fungal cell wall like chitin or the chitin monomer *N*-acetylglucosamine can also induce chitinase gene expression (Kubicek *et al.*, 2001). Glycoproteins (e.g. lectins) located in the host’s cell wall induce coiling of the mycoparasite around host hyphae (Inbar & Chet, 1994). Both secretion of hydrolytic enzymes and host attachment/coiling therefore can be assumed to be induced responses triggered by host-derived molecules. Despite the significance of recognition in the interaction of fungal pathogens with their plant, animal, human or fungal hosts, there is only limited knowledge about receptors for host-derived signals. Intracellular signal transduction pathways are considered to regulate the expression of pathogenicity-related genes in response to the host. Recently, examination of these pathways in *Trichoderma* revealed the involvement of heterotrimeric G proteins in sensing of host signals and in activating mycoparasitic host attack (Rocha-Ramirez *et al.*, 2002; Reithner *et al.*, 2005; Zeilinger *et al.*, 2005; Mukherjee *et al.*, 2004, 2007). G protein signalling is a co-action of seven-transmembrane receptors (G-protein-coupled receptors, GPCRs), heterotrimeric G proteins and an effector, where GPCRs play an essential role in the recognition of extracellular signals (Neer, 1995). Representing one of the largest protein families found in nature (Lander *et al.*, 2001), GPCRs do not share significant sequence similarity but have a common domain structure consisting of seven transmembrane helices connected by intra- and extracellular loops (Dohlman *et al.*, 1991). Binding of a ligand to the receptor leads to a conformational change within the GPCR resulting in the activation of the G protein (Gutkind, 1998). However, little information is available on the characteristics and functions of fungal GPCRs other than pheromone receptors (e.g. Hagen *et al.*, 1986; Blumer *et al.*, 1988; Tanaka *et al.*, 1993; Chang *et al.*, 2003; Kim & Borkovich, 2004; Xue *et al.*, 2006; Hsueh *et al.*, 2009) and the role of these receptors in fungal pathogenicity is largely unknown. In the basidiomycete human pathogen *Cryptococcus neoformans* the *Cpr*a pheromone receptor was shown not only to be required for mating but also to play a significant role in virulence (Chang *et al.*, 2003); in addition, the amino-acid-sensing GPCR Gpr4 was reported to be important for both mating and capsule production (Xue *et al.*, 2006). Recent analysis of *Aspergillus fumigatus* GprC and GprD, which show sequence similarity to the Gpr1p glucose receptor from *Saccharomyces cerevisiae*, revealed that both receptors are essential regulators of colony growth, hyphal morphogenesis and virulence (Gehrke *et al.*, 2010). In the plant pathogen *Magnaporthe grisea*, *pth11* mutants lacking a CFEM-domain-containing seven-transmembrane protein were non-pathogenic due to a defect in appressorium differentiation (DeZwaan *et al.*, 1999). We recently identified more than 50 putative GPCRs in the genome of *Trichoderma reesei* (Brunner *et al.*, 2008) and, based on this analysis, isolated four genes from the mycoparasite *Trichoderma atroviride* encoding seven-transmembrane receptors of the cAMP-receptor-like (CRL) class.

In this study, we explore possible roles for the CRL protein Gpr1 of *T. atroviride* in order to better understand the signalling mechanisms that govern the mycoparasitic interaction with the host fungus. To this end, we isolated target genes of the signalling pathway(s) activated by Gpr1 and showed that *gpr1*-silenced transformants were avirulent in confrontation assays due to their inability to attach to and attack the host. Further analysis revealed that mycoparasitism-relevant processes such as chitinase and protease gene expression and attachment to host hyphae are governed by a functional Gpr1 protein. To our knowledge, Gpr1 is the first fungal member of the CRL class with pathogenicity-related functions and the first functionally characterized receptor of a mycoparasitic fungus. Our data highlight the fundamental role of Gpr1 during sensing of environmental signals and transduction to intracellular regulatory targets during the antagonist–host interaction.

## Methods

### 

#### Strains and culture conditions.

*Trichoderma atroviride* strain P1 (ATCC 74058; teleomorph *Hypocrea atroviridis*) was used for this study; it was maintained on potato dextrose agar (PDA) at 28 °C until sporulation. *gpr1*-silenced transformants were generated and purified by three rounds of single-spore isolation as described previously (Brunner *et al.*, 2008). Due to inhomogeneous growth and conidiation, transformant *gpr1* sil-2 was further purified by an additional round of single spore isolation, resulting in mutant gpr1 sil-2.1. Analysis of the silencing levels of the transformants was performed by real-time RT-PCR as described below using primers gpr1For and gpr1Rev (Table 1). *sar1* was used as reference gene as described previously (Brunner *et al.*, 2008). The transformants were maintained on PDA supplemented with 200 µg hygromycin B ml^−1^. *Escherichia coli* JM 109 was the host for plasmid amplification and was grown as described by Sambrook *et al.* (1989).

**Table 1.** Oligonucleotides used in this study

Gene/primer	Forward primer (5′ to 3′)	Reverse primer (5′ to 3′)
**Primer pairs used for quantification of transcripts by real-time RT-PCR**		
*gpr1*	TTGATCCAGACCTTCATGCCAGC	CATAAAAGGCCGCGACACGAA
*nag1*	TGTCCTACAGCCTCTGCTGCAAAAGTTC	CATCTCCTCACAGACAAGCGGTGAAAG
*ech42*	CGCAACTTCCAGCCTCAGAACC	TCAATACCATCGAAACCCCAGTCC
*prb1*	CGCACTGCTTCCTTCACCAACT	TTTCACTTCATCCTTCGCTCCA
*sar1*	CTCGACAATGCCGGAAAGACCA	TTGCCAAGGATGACAAAGGGG
*act1*	GCACGGAATCGCTCGTTG	TTCTCCACCCCGCCAAGC
128908	TTCGTCGTTATTGCGTCCAC	GGCAGCCTTCTTTCCATACC
90851	TCGCCGATTATCAGAAGG	CGCATACAGAGCTGGATTG
146614	TTCTCGGCTCTTGGGAC	CTCTTGCTGCTTGACTTGTG
132043	AAGACGACGACATCCGAGAC	TGGAATCATTGCCGAACC
142851	TGGTGGTGACCGCGTTACAG	TGGCGTTGAGCACTCCGTTG
137004	ATAATGCCTCCGCCTTCC	CCGACTTGCCCAAATAGC
142792	AAAGGGGCCATGTCTATCAA	GAAAAGGCAACTTCCTCAAA
160894	AAAGGAAGCGGAAAGAAGC	GGAAATGAACATCACCGACC
145570	ATTCGCCGAGGATGAGAG	CGTTGGGGTTTGACTGAG
133072	CACCGCTGCTGACAACAAGG	GCCAGAAGGGCAGCAATGAC
159687	TCGCGTCCCATGATACC	GGAAGTCGTTGAGCACCAG
138324	AACAGCGGATGGGACACG	GGGAGATACCAAAGGAGGGAC
133633	CGAGCAAGTCATCAAGGTG	TGGATAGGCTGCCAAAGTAG
142538	TGCGAAGACGATCCTAGAC	CACAGACCAATCCAAACG
129518	GACACTTCCAGTCCAGCATC	GGGAGGCGATTTGGTTAC
148150	CATAGCAGATGGCTCGTG	GCGACAAAGTTCTGGTGC
**Oligonucleotides used for gene amplification**		
tga1sfi	TGGCCATTACGGCCATGGGTTGCGGAATGTCTACAG	AGGCCGAGGCGGCCGCTAAATGAGACCGCATAAACG
tga2sfi	TGGCCATTACGGCGATGTGCTTCGGGGCTC	AGGCCGAGGCGGCCGTTATAGTATTAACTTTTTGAGG
tga3sfi	TGGCCATTACGGCCATGGGCGGCTGCATGAGC	AGGCCGAGGCGGCCGTTAAAGAATACCCGAATC
tga1dual	TGGCCATTACGGCCAGATGGGTTGCGGAATGTCTAC	GAGGCCGAGGCGGCCATGAGACCGCATAAACGAAGG
tga2dual	TGGCCATTACGGCCCAATGTGCTTCGGGGCTC	GAGGCCGAGGCGGCCAGTATTAACTTTTTGAG
tga3dual	TGGCCATTACGGCCCCATGGGCGGCTGCAT	GAGGCCGAGGCGGCCAGAATACCCGAATCC
tgpcr1sfi	TGGCCATTACGGCCATGGCCGGAGGACTCTCAGAGG	TGGCCGAGGCGGCCACTGCCTGCTCTGGAATTTCTGC
SdMGpr1	GATACCGTCGAGCGCCAAC	CAGGTGATAGGCATGGCCGT

For investigating the expression of mycoparasitism-related genes in liquid media, *T. atroviride* was grown in synthetic medium (SM), containing 2 % glycerol as carbon source, as described previously (Brunner *et al.*, 2003). After 36 h the mycelia were transferred to SM containing 1 % (w/v) of either *N*-acetylglucosamine or colloidal chitin.

For determination of growth rates, fungi were inoculated at the centre of PDA plates and the colony diameters recorded every 24 h. Conidia were quantified by plating a freshly harvested and filtered spore suspension containing 1×10^6^ conidia onto PDA plates and counting the conidia produced after 3 days of incubation at 28 °C. To test the influence of exogenous cAMP, 5 mM cAMP (Sigma) was added to PDA as this concentration was previously shown to result in the most pronounced effect on coiling in *T. atroviride* (Omero *et al.*, 1999; Zeilinger *et al.*, 2005). For determination of fungal biomass after growth in liquid culture, mycelial dry weight was measured.

#### DNA and RNA procedures.

Standard molecular techniques were performed according to Sambrook *et al.* (1989); DNA and RNA isolation was carried out as described by Peterbauer *et al.* (1996). For standard PCR amplification, recombinant *Taq* polymerase was used.

For cDNA preparation, RNAs were incubated with DNase I (1 U per µg RNA) to remove remaining chromosomal DNA. First-strand cDNA synthesis was carried out with an oligo(dT)_18_ primer (0.5 µg µl^−1^) and a random hexamer primer (0.2 µg µl^−1^), 1 µg total RNA and Revert Aid H Minus M-MuLV reverse transcriptase (200 U µl^−1^).

#### Real-time RT-PCR.

All quantifications were performed with the following PCR programme: initial denaturation for 180 s at 95 °C, 50 cycles of 95 °C for 20 s, 60 °C for 20 s and 72 °C for 20 s on an Eppendorf *realplex^2^S* Mastercycler using the IQ SYBR Green Supermix (Bio-Rad) and 25 µl assays with standard MgCl_2_ concentration (3 mM) and with final primer concentrations of 100 nM each (Table 1). All assays were carried out in 96-well plates covered with optical tape. In both the parental strain and the *gpr1* mutants, *sar1* turned out by geNorm (Vandesompele *et al.*, 2002) analysis to be the most stable internal control gene for cultivation on solid media, whereas *act1* was the best reference gene for cultivations in liquid media (Brunner *et al.*, 2008). PCR efficiency was determined from a single-tube reaction set-up as described by Tichopad *et al.* (2003), and the expression ratio was calculated according to the equation published by Pfaffl (2001). All samples were analysed in three independent experiments with three replicates in each run.

#### Microscopy.

Microscopic studies were mainly performed as described by Lu *et al.* (2004). Briefly, 500 µl PDA was spread onto glass slides, inoculated, and incubated on a moistened filter paper at 28 °C in a Petri dish sealed with Parafilm. *T. atroviride* and *R. solani* were inoculated on opposite sides of the glass slides. After 48−72 h the fungal hyphae were examined with a Leitz Aristoplan microscope and pictures were taken using an Olympus DP 10 camera.

Confocal microscopic studies were performed using the membrane-sensitive red dye FM4-64 (Invitrogen), which stains the membranes of intact hyphae and the complete hyphal compartment of dead hyphal fragments due to the permeability of the cell wall of dead cells. For sample preparation, *T. atroviride* and *R. solani* were inoculated on opposite sides of a PDA plate and incubated for 48–72 h at 28 °C. For investigation of the fungal hyphae, the ‘inverted agar block method’ was used as described by Hickey et *al*. (2005). Briefly, a ~10 mm^2^, 5 mm thick block of agar from the region of the colony to be imaged was inverted onto a droplet of water containing the FM4-64 dye upon a glass coverslip. Excess medium was removed using a filter paper. Imaging was carried out with an inverted Nikon TE-2000 microscope equipped with a C1 confocal system. Images were taken with the EZ C1 software (Nikon) and digitally processed using the software Image J (http://rsb.info.nih.gov/ij/).

#### Antagonistic assays.

Plate confrontation assays were performed as described previously (Lorito *et al.*, 1996; Zeilinger *et al.*, 1999). Agar plugs of *Trichoderma* were cultivated together with a host fungus on PDA plates covered with a cellophane membrane at 28 °C. When the mycelia of the two fungi came into contact, mycelium of *Trichoderma* from the confrontation zone was harvested for RNA extraction.

#### Quantitative 6-pentyl-α-pyrone (6-PP) analysis.

Quantification of 6-PP from PDA plates was performed as described previously (Reithner *et al.*, 2005).

#### Measurement of intracellular cAMP levels.

Mycelia from strains grown for 72 h on PDA were obtained as described previously (Zeilinger *et al.*, 2005). The cAMP content was determined using the Direct cAMP Enzyme Immunoassay kit (Sigma) according to the manufacturer's instructions. The cAMP levels were related to protein concentrations (measured by the Bio-Rad protein assay) of the samples and were expressed as means±sd for three independent experiments.

#### cAMP-dependent protein kinase (PKA) activity assay.

After growing *T. atroviride* for 72 h on PDA, mycelia were harvested, and 50 mg mycelium was ground in liquid nitrogen and homogenized three times for 10 s in 200 µl lysis buffer (20 mM Tris/HCl, pH 7.4, 1 mM EDTA, protease inhibitors). The homogenate was centrifuged at 30 000 ***g*** for 2 h at 4 °C. The supernatants were collected and the protein concentration determined using Bradford reagent (Bio-Rad). PKA activity of 0.5 µg crude protein from three replicate samples obtained from independent cultivations was assayed using the Assay Designs Non-radioactive PKA Kinase Activity Assay kit (Assay Designs, Stressgen) with serially diluted active PKA serving as a positive control. The final colour development was stopped after 30 min and the colour intensity was measured by determining the absorbance at 450 nm.

#### Dual-membrane yeast two-hybrid assay.

A split ubiquitin-based yeast two-hybrid assay was performed to test Gpr1–Gα interactions. Vectors and yeast strains included in the DUALmembrane kit 3 (Dualsystems Biotech) were used. *gpr1* full-length cDNA was amplified with primers tgpcr1sfiF and tgpcr1sfiR (Table 1) and introduced into vector pBT3-STE, thereby resulting in a fusion of the C-terminal half of ubiquitin Cub along with the transcription factor LexA-VP16 to the C-terminus of Gpr1. The mutated N-terminal half of ubiquitin NubG was fused to the N-terminus (vector pPR3 N) of *tga1*, *tga2* and *tga3* full-length cDNAs using primers tga1sfiF/tga1sfiR, tga2sfiF/tga2sfiR and tga3sfiF/tga3sfiR, respectively, and to the C-terminus (vector pPR3-STE) using primers tga1dualF/tga1dualRev, tga2dualF/tga2dualRev and tga3dualF/tga3dualRev (Table 1). The correct cDNA sequences were verified by sequencing the respective inserts. The Cub vector bearing the *gpr1* cDNA was co-transformed with each of the NubG vectors encoding one of the three G protein α subunits into yeast strain NMY51. Substitution of proline by leucine at position 344 (L344P) of Gpr1 was done by introducing a point mutation at position 1031 in the open reading frame of the *gpr1* gene. The mutation was introduced using phosphorylated primers SdMGpr1F and SdMGpr1R (Table 1) and the Phusion Site-directed mutagenesis kit (Finnzymes). The mutated *gpr1* full-length cDNA was introduced into vector pBT3-STE. The mutation was verified by sequencing the respective insert. The Cub vector bearing the mutated *gpr1* cDNA was co-transformed with each of the NubG vectors encoding one of the three Gα subunits into yeast strain NMY51.

Interactions were confirmed by growing transfected yeast on media lacking histidine or histidine and adenine and by measurement of β-galactosidase activity as a quantitative measure of the interaction. To prove that Gpr1 is actually expressed, a functional assay using the wild-type ubiquitin half NubI fused to the endogenous endoplasmic reticulum protein Alg5 as an interaction partner of Gpr1 was performed according to the manufacturer’s instructions.

#### Suppression subtractive hybridization (SSH).

The *T. atroviride* parental strain and the *gpr1*-silenced transformant sil-8 were separately grown on PDA. Total RNA was extracted from the harvested mycelia and subtractive cDNA libraries were constructed using the PCR-Select cDNA subtraction kit (Clontech). cDNA from the transformant strain (driver) was subtracted from cDNA of the parental strain (tester). The cDNAs obtained were ligated into vector pGEM-T (Promega) and transformed into *E. coli* JM109. The presence of inserts was confirmed by PCR in 120 randomly collected clones. A total of 30 independent clones were selected for sequencing on the basis of their different insert sizes. The sequences obtained were used as a query to perform a tblastn search against the *T. atroviride* genome database (http://genome.jgi-psf.org/Triat1/Triat1.home.html; Kubicek *et al.*, 2011). The full-length sequences of the respective hypothetical proteins were retrieved from the genome database and used to perform a blastp search of the NCBI database (http://www.ncbi.nlm.nih.gov/).

## Results

### The *T. atroviride* genome contains four genes encoding seven-transmembrane proteins of the cAMP-receptor-like family

Recently, we reported on the screening of the *T. reesei* genome for the presence of putative GPCRs. Based on these results, four *T. atroviride* genes (*gpr1*, *gpr2*, *gpr3* and *gpr4*) encoding members of the cAMP-receptor-like (CRL) family were isolated by screening a genomic library (Brunner *et al.*, 2008). As the genome sequence of *T. atroviride* was released recently, an additional *in silico* exploration of its database (Kubicek *et al.*, 2011; http://genome.jgi-psf.org/Triat1/Triat1.home.html) for members of the CRL family could be performed. No additional family members were found by a blastp search using the protein sequences of Gpr1, Gpr2, Gpr3 and Gpr4 as query. In addition, the previously isolated *gpr1*, *gpr2*, *gpr3* and *gpr4* genes and their deduced amino acid sequences were completely consistent with their respective counterparts in the genome database, in which Gpr1 corresponds to protein ID160995, Gpr2 to ID50902, Gpr3 to ID83166 and Gpr4 to ID81233. Evaluation of the protein sequences with the tmhmm algorithm (Krogh *et al.*, 2001) confirmed the presence of seven transmembrane regions and a topology typical for members of the CRL family, with five transmembrane domains at the N-terminal end, a long third intracellular loop and two helices next to the C-terminus.

Further characterization of the *gpr1* gene revealed an open reading frame of 1446 bp harbouring two introns of 80 bp and 58 bp, respectively, which encodes a protein of 48.8 kDa. Gpr1 shows 52 % amino acid sequence identity to Gpr2, 28 % to Gpr3 and 30 % to Gpr4; 42 % sequence identity was found with *Neurospora crassa* GPR-2 and GPR-3, and 25 % with *N. crassa* GPR-1, the only CRL protein from an ascomycete functionally characterized up to now (Krystofova & Borkovich, 2006).

**Fig. 1.**
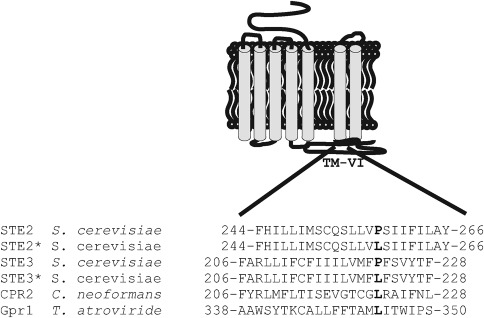
Amino acid sequences of transmembrane domain 6 (TM-VI) of *S. cerevisiae* Ste2 and Ste3, *C. neoformans* Cpr2 and *T. atroviride* Gpr1. Substitution of the conserved proline for leucine in Ste2 and Ste3 results in constitutive activation (*) of the respective receptors (Baldwin, 1993). The leucine resulting in constitutive activity of Cpr2, Ste2 and Ste3 is in bold.

As G protein signalling is a co-action of a GPCR and a heterotrimeric G protein, we investigated whether Gpr1 directly interacts with any of the three Gα subunits of *T. atroviride.* When employing the split ubiquitin membrane-based yeast two-hybrid system (Stagljar *et al.*, 1998), no physical interaction could be observed between Gpr1 and Tga1, Tga2 or Tga3 (data not shown). A constitutive activity of the receptor which results in its continuous dissociation from the interacting Gα protein may result in a failure to detect their direct interaction. Constitutive receptor activity was described to be caused by a substitution of a conserved proline by a leucine in the sixth transmembrane domain (TM-VI) (e.g. Baldwin, 1993; Hsueh *et al.*, 2009). To test this possibility, we carefully determined the exact position of TM-VI of *T. atroviride* Gpr1 using the tmhmm algorithm. A comparison of the amino acid sequence of TM-VI of Gpr1 with the native constitutively active Cpr2 receptor of *C. neoformans* and mutant versions of *S. cerevisiae* Ste2 and Ste3 in which proline was artificially replaced by leucine (Konopka *et al.*, 1996; Hsueh *et al.*, 2009) showed that Gpr1 does not contain a proline in the conserved position but instead a leucine (Fig. 1). To assess if the missing interaction between Gpr1 and the Gα subunits in the split ubiquitin membrane-based yeast two-hybrid system was caused by a constitutive activity of Gpr1, a Gpr1^L344P^ allele was generated. Again, no physical interaction with Tga1, Tga2 or Tga3 could be demonstrated (data not shown).

### Phenotypic characterization of *gpr1*-silenced transformants

As several attempts to obtain *gpr1* gene deletion mutants failed, a silencing approach was performed in order to functionally characterize the Gpr1 receptor. Four mitotically stable strains (*gpr1* sil-2.1, *gpr1* sil-6, *gpr1* sil-7 and *gpr1* sil-8) bearing ectopic integration of the silencing vector pSilent2-gpr1 (Brunner *et al.*, 2008) were randomly chosen for further studies. They showed silencing levels of the *gpr1* gene of 70–80 % (Table 2).

**Table 2.** Characteristics of *gpr1*-silenced transformants

	Parental strain	*gpr1* sil-2.1	*gpr1* sil-6	*gpr1* sil-7	*gpr1* sil-8
Level of *gpr1* silencing (%)*†	0	81±2.9	70±2.7	75±2.5	80±2.3
Growth rate (mm per 10 h)†	13±0.26	5.5±0.16	4.9±0.20	5.4±0.16	5.6±0.26
No. of spores (×10^8^)†	6.3±0.4	24.3±10.2	16.8±1.4	22.2±1.7	16.7±5.1
cAMP (pmol mg^−1^)†	20.24±1.57	4.65±0.42	4.35±0.39	7.25±0.67	6.80±0.54
PKA activity (%)†‡	100	89.3±3.4	93.3±2.8	90.2±1.9	66.2±6.7

* *gpr1* silencing levels were determined as described by Brunner *et al.* (2008) using *sar1* as reference gene.

† Results are the means±sd of three independent experiments.

‡ PKA activities are given as relative values for the transformants compared to the activity of the parental strain.

On solid medium, the transformants exhibited reduced growth rates and colony sizes and produced approximately threefold increased amounts of conidia compared to the parental strain (Table 2). The intracellular cAMP levels of the *gpr1*-silenced transformants were slightly reduced compared to the parental strain, and their PKA activities also differed only slightly (Table 2).

### Identification of Gpr1 target genes

To identify genes being positively regulated by the signalling pathway(s) involving Gpr1, we applied suppression subtractive hybridization (SSH) using transformant *gpr1* sil-8. blast searches of the isolated sequences in the *T. atroviride* genome database resulted in the identification of 20 different genes. Among those, two genes (IDs 159687 and 128908) were represented by different ESTs nine and three times, respectively. The proteins encoded by the 20 genes identified were retrieved from the *T. atroviride* genome database. They showed similarity to proteins involved in diverse functions such as protein biosynthesis and folding, electron transport, carbohydrate metabolism, cell wall components, and proteins with unknown function (Table 3).

**Table 3.** *T. atroviride* genes isolated by SSH

Clone corresponds		Domain/superfamily	Best blastp match	E-value
to ID	Annotated as			
159687		Cyclin superfamily	Hypothetical protein CHGG_10926 (*Chaetomium globosum* CBS 148.51); pho85 cyclin-7 (*Ajellomyces capsulatus* H143, 3e–38)	5e–41
133072	Predicted SSCP		Hypothetical protein CHGG_01484 (*Chaetomium globosum* CBS 148.51)	5e–17
142538	Molecular chaperone bip1	HSP70	Predicted protein (EEU40459.1, *Nectria haematococca* mpVI 77-13-4)	0.0
128908	Candidate cytochrome P450	CypX superfamily, p450	Hypothetical protein FG01959.1 (*Gibberella zeae* PH-1)	0.0
142851		Chaperonin-like superfamily	Predicted protein (EEU46541.1, *Nectria haematococca* mpVI 77-13-4)	0.0
142792		Ribosomal_L7Ae superfamily	40S ribosomal protein S12 (*Gibberella zeae* PH-1)	7e–67
133633		GAL4	Predicted protein (EEU41863.1, *Nectria haematococca* mpVI 77-13-4)	0.0
129518	Candidate α-glycosyltransferase related to glycogenin	GT8_glycogenin	Unnamed protein product (XP_001911342.1, *Podospora anserina*)	1e–128
41949	Related to GPI17/PIG-S component of GPI transamidase complex	PIG-S superfamily	Hypothetical protein CHGG_10895 (*Chaetomium globosum* CBS 148.51)	0.0
138324	Candidate β-glycosidase related to β-*N*-acetylhexosaminidase	Glycol_hydro_3 superfamily	β-*N*-Acetylglucosaminidase Nag3 (*Hypocrea virens*)	0.0
127886	Electron transporter	UBQ superfamily	Ubiquitin (*Ajellomyces dermatidis* ER-3)	3e–123
148150			Hypothetical protein NECHADRAFT_78962 (*Nectria haematococca* mpVI)	0.0
90851		Pro-kuma_activ superfamily, COG4934	Putative alkaline serine protease AorO (*Talaromyces stipitatus* ATCC 10500)	7e–159
146614			Hypothetical protein FG10132.1 (*Gibberella zeae* PH-1); esdC *Aspergillus nidulans*, 2e–57)	7e–90
132043			Hypothetical protein FG09412.1 (*Gibberella zeae* PH-1)	0.0
137004		DUF2467 superfamily	Conserved hypothetical protein (XP_002481139.1, *Talaromyces stipitatus* ATCC 10500)	6e–52
146236		EF1_alpha	Translation elongation factor 1a (*Hypocrea jecorina*)	0.0
129811		EF1G superfamily	Elongation factor 1-gamma (*Neurospora crassa* OR74A)	2e–154
160894			Hypothetical protein FG10108.1 (*Gibberella zeae* PH-1)	6e–08
145570		Uricase	Uricase (*Tolypocladium inflatum)*	7e–136

**Fig. 2.**
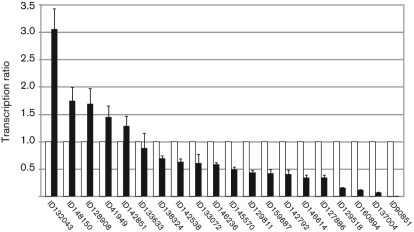
Relative transcription ratios of the 20 genes retrieved by SSH in the *gpr1* sil-8 transformant (black bars) in comparison to the parental strain (white bars) upon cultivation on PDA. mRNA levels were determined by real-time RT-PCR using *sar1* as reference gene. Samples of the parental strain were assigned the value 1 for each individual gene. Results are means±sd (*n* = 3).

For 14 of the 20 genes, significantly lower mRNA levels in the *gpr1*-silenced transformant than in the parental strain upon cultivation on PDA could be verified by real-time RT-PCR (Fig. 2). These genes encoded proteins with IDs 159687 (protein of the cyclin-like superfamily), 129518 (candidate α-glycosyltransferase), 138324 (candidate β-glycosidase), 90851 (putative protease), 146614 (hypothetical protein with similarity to *Aspergillus nidulans* EsdC), 142792 (ribosomal protein), 145570 (hypothetical protein with similarity to uricase), 142538 (molecular chaperone Bip1), 127886 (electron transporter), 133072 (small secreted cysteine-rich protein, SSCP, belonging to cluster 1, Kubicek *et al.*, 2011), 129811 (putative elongation factor), 146236 (putative translation elongation factor), 137004 (protein of unknown function) and 160894 (protein of unknown function). Two genes (IDs 133633 and 142851) showed similar mRNA levels in the *gpr1*-silenced transformant and the parental strain, and four genes (IDs 128908, 148150, 41949 and 132043) even exhibited enhanced transcription compared to the parental strain, suggesting that these are false positives.

When comparing the expression of the 14 genes verified as targets of Gpr1 upon cultivation in the absence of a host to cultivation in the presence of *R. solani,* only the SSCP (ID 133072) turned out to be induced (~2.5-fold) upon mycoparasitism in the parental strain. However, none of these genes was induced by the presence of the host in the *gpr1*-silenced transformant (data not shown).

### *gpr1*-silenced transformants are affected in their antagonistic activity

**Fig. 3.**
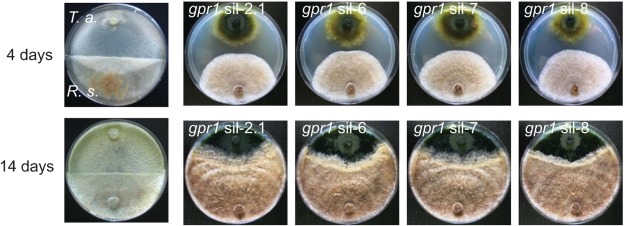
Plate confrontation assays of *T. atroviride* parental strain and the *gpr1*-silenced transformants with *R. solani*. Pictures were taken 4 and 14 days after inoculation of the two fungi on opposite sides of the plate.

To investigate the role of the Gpr1 receptor during the interaction with a living host fungus, plate confrontation assays against *R. solani* were performed. The parental strain began to overgrow and lyse the host fungus after ~4 days, whereas the *gpr1*-silenced transformants were completely avirulent, i.e. unable to attack and parasitize the host even after 14 days (Fig. 3). Similar results were obtained when *S. sclerotiorum* or *B. cinerea* were used as hosts (data not shown).

During the early stages of the confrontation (e.g. at day 4, Fig. 3) a slight growth inhibition of the host fungus by the *gpr1*-silenced transformants was observed, suggesting the involvement of an antibiosis-like mechanism. However, quantification of 6-pentyl-α-pyrone (6-PP), one of the most important antifungal metabolites produced by *T. atroviride* (Claydon *et al.*, 1987), in the parental strain [104.74±17.97 mg 6-PP (g mycelial dry weight)^−1^] and the *gpr1*-silenced transformant sil-8 [0.77±0.39 mg 6-PP (g mycelial dry weight)^−1^] revealed drastically reduced amounts secreted by the transformant.

**Fig. 4.**
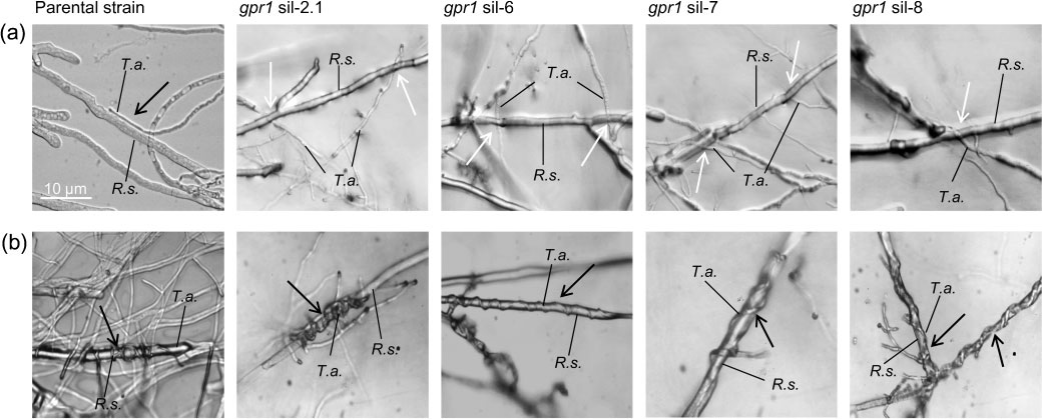
Microscopic examination of the interaction of the *T. atroviride* parental strain and the *gpr1*-silenced transformants with *R. solani* in the absence (a) or presence (b) of 5 mM cAMP. The co-cultures were incubated until contact between the two fungi. Attachment to and growth alongside the host and coiling around the host hyphae in the presence of cAMP is indicated by black arrows; hyphae growing past each other without mutual recognition are indicated by white arrows.

Microscopic investigations of the confrontation zone with *R. solani* revealed that the parental strain recognized the host, as indicated by an attachment to and growth of the mycoparasite alongside the host's hyphae, which typically precedes coiling around the host. In contrast, the *gpr1*-silenced transformants did not attach to the host (Fig. 4a). As cAMP was repeatedly shown to promote mycoparasitic coiling of *Trichoderma* (e.g. Omero *et al.*, 1999), exogenous cAMP was added to the plate confrontation assay. Interestingly, cAMP was able to restore attachment to and subsequent coiling around host hyphae in the *gpr1*-silenced transformants (Fig. 4b), although it was unable to restore mycoparasitic overgrowth and the hypersporulating phenotype.

These results suggest that Gpr1 governs attachment of *T. atroviride* to host hyphae by transducing the signal via the cAMP pathway, which is similar to the previously described functions of the Tga3 Gα subunit (Zeilinger *et al.*, 2005).

### *gpr1* gene silencing affects the expression of mycoparasitism-related genes

**Fig. 5.**
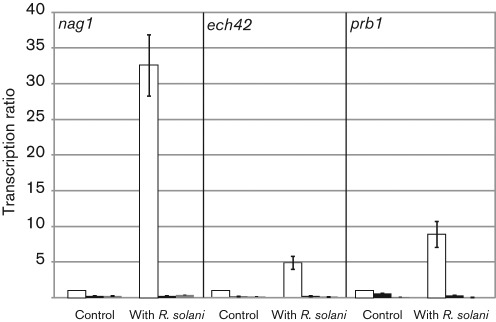
Relative transcription ratios of the chitinase-encoding genes *nag1* and *ech42,* and the protease-encoding gene *prb1* in transformants *gpr1* sil-7 (grey bars) and sil-8 (black bars) and the parental strain (white bars) during confrontation with *R. solani*. Samples were taken from a control in which the parental strain and the *gpr1*-silenced transformants were grown alone and directly after contact with *R. solani* as host and subjected to real-time RT-PCR using *sar1* as reference gene. The control sample of the parental strain was arbitrarily assigned the value 1. Results are means±sd (*n* = 3).

Besides attachment to and coiling around host hyphae, the formation of infection structures and production of hydrolytic enzymes such as chitinases and proteases play a major role during *Trichoderma* mycoparasitism (Geremia *et al.*, 1993; Kubicek *et al.*, 2001). To determine whether the avirulent phenotype of *gpr1-*silenced transformants correlates with altered expression levels of selected genes encoding cell-wall-degrading enzymes, we analysed the transcription of the chitinase-encoding *nag1* and *ech42* genes and the protease-encoding *prb1* gene during confrontation with *R. solani*. As the four *gpr1*-silenced transformants tested showed the same behaviour in plate confrontation assays and in host attachment and coiling, *gpr1* sil-7 and sil-8 were selected for analysis of enzyme production. In the parental strain, all three genes tested showed a basal level of transcription in the absence of a fungal host and were significantly induced upon contact with *R. solani* (Fig. 5). In the *gpr1-*silenced transformants confronted with *R. solani* no transcriptional induction of the three tested genes over the basal level could be observed. These results suggest that the Gpr1 receptor is essential for inducing the expression of mycoparasitism-related genes by the presence of a living host fungus.

**Fig. 6.**
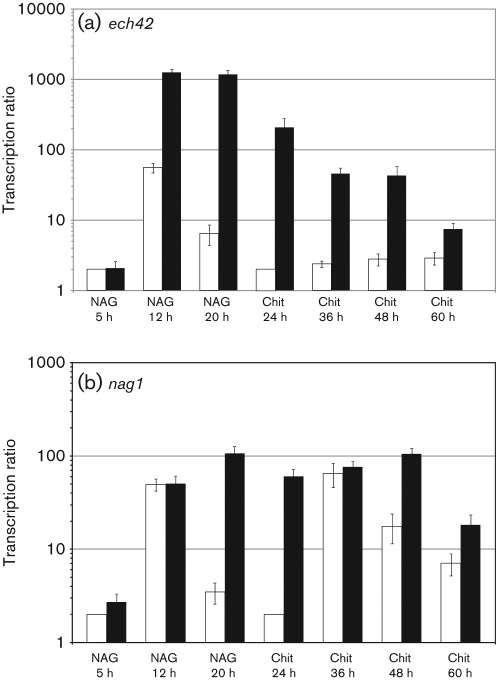
Relative transcription ratios of the chitinase-encoding genes *ech42* (a) and *nag1* (b) in transformant *gpr1* sil-8 (black bars) and the parental strain (white bars). Real-time RT-PCR was performed 5, 12 and 20 h after transfer of the mycelia to liquid growth medium containing 1 % *N*-acetylglucosamine (NAG), and 24, 36, 48 and 60 h after transfer to medium containing 1 % colloidal chitin (Chit), using *act1* as reference gene. Samples of the parental strain at NAG 5 h and Chit 24 h were arbitrarily assigned the value 1. To all values 1 was added to allow illustration on a logarithmic scale. Results are means±sd (*n* = 3).

In order to find out if the *gpr1-*silenced transformants are unable to sense the living host or if they have a general defect in the production of cell-wall-degrading enzymes, we analysed *ech42* and *nag1* gene transcription in liquid cultures (Fig. 6). Both genes were induced by *N*-acetylglucosamine and colloidal chitin in both the parental strain and the *gpr1-*silenced transformant tested; the latter even showed enhanced *nag1* and *ech42* mRNA levels. When analysing chitinase enzyme activities, a similar picture was obtained (data not shown).

**Fig. 7.**
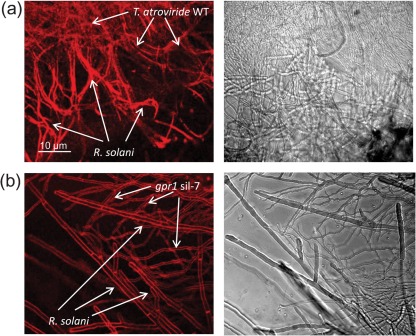
Confocal microscopic studies of the interaction of the *T. atroviride* parental strain (WT, a) and the *gpr1*-silenced transformant sil-7 (b) with *R. solani*. Co-cultures were incubated until contact between the two fungi and pictures (left, confocal images; right, bright-field images) were taken after staining with FM4-64. Arrows indicate hyphae of the *T. atroviride* parental strain and the *gpr1*-silenced transformant which are in contact with *R. solani.* Upon interaction with the parental strain (a), hyphae of *R. solani* are lysed, resulting in staining of the complete hyphal compartment of the dead hypha. Upon confrontation with the *gpr1*-silenced transformant (b), hyphae of *R. solani* remain completely intact, reflected by the stained intact hyphal membrane.

The ability of *gpr1*-silenced transformants to lyse and kill living host hyphae was also investigated by confocal microscopy using the red-fluorescing dye FM4-64. This dye stains the membranes of intact hyphae and the complete hyphal compartment of dead hyphal fragments. Whereas the hyphae of *R. solani* interacting with the *T. atroviride* parental strain appeared completely red, proving their lysis by cell-wall-degrading enzymes secreted by *Trichoderma*, host hyphae were completely undamaged upon confrontation with the *gpr1*-silenced transformant sil-7 (Fig. 7).

Taken together, these results suggest that silencing of *gpr1* results in an inability to respond to the presence of *R. solani* and to lyse and kill the living host fungus.

## Discussion

The Gα subunits Tga1 and Tga3 were previously shown to play crucial roles in signalling in the mycoparasite *T. atroviride*. They govern vegetative growth as well as mycoparasitism-relevant processes such as host attachment and coiling, and the production of chitinases and antifungal metabolites (Rocha-Ramirez *et al.*, 2002; Reithner *et al.*, 2005; Zeilinger *et al.*, 2005). Because of this substantial role of G protein signalling, we were interested in the respective receptors acting upstream.

Despite the presence of numerous GPCR-encoding genes in fungal genomes, only a few of them have been functionally characterized. We recently reported on the isolation of four genes of *T. atroviride* which encode putative GPCRs of the CRL family (Brunner *et al.*, 2008). This class of fungal receptors is defined via the structural similarity of its members to the cAR cAMP receptors of *Dictyostelium discoideum*, whereas it is absent in the genomes of the ascomycete yeasts *Saccharomyces cerevisiae* and *Schizosaccharomyces pombe* (Lafon *et al.*, 2006; Li *et al.*, 2007). The first CRL protein functionally characterized in an ascomycete fungus was *N. crassa* GPR-1, which is required for female sexual development (Krystofova & Borkovich, 2006) and which is the orthologue of *T. atroviride* Gpr3.

Despite their similarity on the sequence and topology level, CRL receptors seem to have diverse functions in different species. Whereas no obvious effects during asexual growth and development were reported for *N. crassa* Δ*gpr-1* mutants (Krystofova & Borkovich, 2006), *T. atroviride gpr1*-silenced transformants exhibited reduced growth and permanent conidiation on solid media. Furthermore, although members of the fungal CRL class exhibit amino acid sequence similarity to the cAMP-sensing receptors cAR1-cAR4 of *D. discoideum*, there is no evidence that extracellular cAMP is sensed via CRL proteins in fungi.

*T. atroviride gpr1-*silenced transformants, similar to Δ*tga3* mutants (Zeilinger *et al.*, 2005) still responded to exogenous cAMP, as its addition resulted in the abrogation of their defect in attaching to and coiling around host hyphae; this makes it unlikely that cAMP is sensed by Gpr1. The finding that cAMP addition was not able to restore mycoparasitic overgrowth of the *gpr1-*silenced transformants suggests that this property is mediated through a cAMP-independent pathway.

In accordance with their defect in attaching to host hyphae, *gpr1-*silenced transformants were unable to attack and lyse host fungi upon direct confrontation (Figs 3 and 7). In addition, other processes known to contribute to *Trichoderma* mycoparasitism, such as the production of cell-wall-degrading enzymes, could not be induced by the living host, suggesting that Gpr1 might be involved in sensing of the host and in activating the signalling cascade(s) resulting in the mycoparasitic attack.

Concerning the production of cell-wall-degrading enzymes, it is interesting that transcription of *nag1* and *ech42* could not be induced in the *gpr1*-silenced transformants upon confrontation with a living host, although chitinase gene transcription, as in Δ*tga3* mutants (Zeilinger *et al.*, 2005), was still inducible by *N*-acetylglucosamine or colloidal chitin in liquid culture. This defect may be due to the fact that *gpr1-*silenced transformants are unable to recognize host hyphae and attach to them, which is a prerequisite for induction of the transcription of certain chitinases such as *nag1* (Zeilinger *et al.*, 1999). *nag1* expression, in turn, was shown to be indispensable for the induction of additional chitinase-encoding genes of *T. atroviride* such as *ech42* (Brunner *et al.*, 2003). In addition, it is worth mentioning that silencing of *gpr1* resulted in a reduced level of constitutively expressed chitinase genes (Fig. 5), which are believed to be responsible for the initial attack of the host cell wall followed by the release of host-derived cell wall degradation products which then act as inducers for the full induction of the chitinolytic enzyme machinery (Kullnig *et al.*, 2000). The isolation of a β-glycosidase showing high similarity to *Trichoderma virens* Nag3 by SSH (Table 3) and the finding that its expression is significantly reduced in the *gpr1-*silenced transformants compared to the parental strain could point to a defect of the transformants in generating the inducer from the living host due to reduced constitutive expression of chitinases.

The gene most frequently isolated by the SSH approach encodes a protein belonging to the cyclin-like superfamily (ID159687) with similarity to the Pho85 cyclin-7 (Pcl7). Investigation of its transcription revealed a 60 % decrease in transformant *gpr1* sil-8 compared to the parental strain (Fig. 2). There is evidence that Pcl7, upon association with the cyclin-dependent kinase Pho85, could be involved in nutrient utilization in fungi, as a Δ*pcl7* mutant of *S. cerevisiae* is unable to use galactose, maltose or lactose as carbon sources, or proline as a nitrogen source (Lee *et al.*, 2000). Interestingly, previous analysis of the carbon utilization properties of the *T. atroviride* *gpr1* sil-8 transformant using Biolog phenotype arrays revealed drastically reduced growth of the mutant on galactose and maltose, and almost no growth on lactose and proline, whereas these were among the best carbon sources for the parental strain (Brunner *et al.*, 2008). These data suggest that Gpr1 could be involved in the utilization of nutrients, possibly by regulating the expression of the Plc7-like cyclin. In addition, it is worth mentioning that in the phytopathogenic fungus *Ustilago maydis* the Pho85/Cdk5 cyclin-dependent kinase was found to control cell-cycle regulation and polar growth of hyphae, both of which are directly related to the pathogenic development (Pérez-Martín & Castillo-Lluva, 2008). Similar processes resulting from the downregulation of cyclin expression could also be one of the reasons for the observed inability of *gpr1*-silenced transformants to undergo the typical mycoparasitism-related morphological changes.

As one of the additional targets of Gpr1, a gene encoding a small secreted cysteine-rich protein (SSCP; ID133072) was identified by the SSH approach. This gene showed an upregulation during mycoparasitism in the parental strain, whereas its expression could not be induced by the host in the *gpr1-*silenced transformants. Recent clustering of SSCPs identified in the *Trichoderma* genomes revealed *T. atroviride* ID133072 to belong to cluster 1, for whose members no function is known yet (Kubicek *et al.*, 2011). Further investigations are warranted to study the role of this SSCP during the antagonistic *Trichoderma*–host interaction and find out in which of the processes contributing to mycoparasitism this protein is involved.

When analysing possible interactions between Gpr1 and the three G protein α subunits of *T. atroviride*, we could not observe any physical interaction between the receptor and Tga1, Tga2 or Tga3. As *gpr1*-silenced transformants and Δ*tga3* mutants (Zeilinger *et al.*, 2005) share several phenotypes such as reduced growth with only few aerial hyphae, continuous sporulation on solid media, defects in attaching to host hyphae, and similar alterations in the expression of cell-wall-degrading enzymes, this result was quite unexpected. Nevertheless, G protein-independent signalling has been assumed for several seven-transmembrane receptors (e.g. Brzostowski & Kimmel, 2001) including those of fungi (e.g. Wang *et al.*, 2010).

Our study revealed a role of the Gpr1 seven-transmembrane receptor in transduction of host-derived signals to intracellular regulatory targets resulting in activation of processes contributing to the mycoparasitic host attack of *T. atroviride*. The fact that the defect of *gpr1-*silenced transformants in attaching to host hyphae could be restored by cAMP prompted us to postulate that Gpr1 regulates infection structure formation by signalling via the cAMP pathway. However, as no direct interaction between Gpr1 and the three Gα proteins could be proven by the approach we used, the possibility that Gpr1 (also) signals in a G-protein-independent manner cannot be ruled out at the moment. Screening for Gpr1- and Tga3-interacting proteins will help to further elucidate the G protein signalling pathways underlying *Trichoderma* mycoparasitism.
